# 2-Benzenesulfonamidobenzoic acid

**DOI:** 10.1107/S1600536809016900

**Published:** 2009-05-14

**Authors:** Abdullah Mohamed Asiri, Mehmet Akkurt, Salman A. Khan, Muhammad Nadeem Arshad, Islam Ullah Khan, Hafiz Muhammad Adeel Sharif

**Affiliations:** a Chemistry Department, Faculty of Science, King Abdul-Aziz University, PO Box 80203, Jeddah 21589, Saudi Arabia; b Department of Physics, Faculty of Arts and Sciences, Erciyes University, 38039 Kayseri, Turkey; c Department of Chemistry, Government College University, Lahore, Pakistan

## Abstract

In the title compound, C_13_H_11_NO_4_S, the dihedral angle between the planes of the benzene ring and the carboxyl group is 13.7 (1)°. The mol­ecular structure contains intra­molecular N—H⋯O and C—H⋯O hydrogen-bonding inter­actions, while the crystal packing is stabilized by C—H⋯O and O—H⋯O hydrogen bonds and C—H⋯π inter­actions. The O—H⋯O hydrogen bonds form a cyclic dimer, with graph-set motif *R* ^2^_2_(8), about a centre of symmetry.

## Related literature

For background to sulfonamide derivatives, see: Sheppard *et al.* (2006 ▶). For similar structures, see: Arshad *et al.* (2009 ▶); Sethu Sankar *et al.* (2002 ▶); Wijeyesakere *et al.* (2008 ▶). For bond-length data, see: Allen *et al.* (1987 ▶). For hydrogen-bond graph-set terminology, see: Bernstein *et al.* (1995 ▶); Etter (1990 ▶).
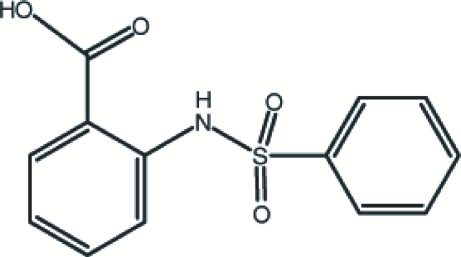


## Experimental

### 

#### Crystal data

C_13_H_11_NO_4_S
*M* *_r_* = 277.30
Monoclinic, 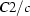

*a* = 27.271 (3) Å
*b* = 8.7223 (9) Å
*c* = 11.0077 (10) Å
β = 106.149 (3)°
*V* = 2515.0 (4) Å^3^
*Z* = 8
Mo *K*α radiation
μ = 0.27 mm^−1^
*T* = 296 K
0.36 × 0.26 × 0.11 mm

#### Data collection

Bruker Kappa APEXII CCD area-detector diffractometer
Absorption correction: none
12048 measured reflections
2989 independent reflections
1824 reflections with *I* > 2σ(*I*)
*R*_int_ = 0.047

#### Refinement

*R*[*F* ^2^ > 2σ(*F* ^2^)] = 0.041
*wR*(*F* ^2^) = 0.105
*S* = 1.00
2989 reflections
173 parameters
H-atom parameters constrained
Δρ_max_ = 0.34 e Å^−3^
Δρ_min_ = −0.25 e Å^−3^

### 

Data collection: *APEX2* (Bruker, 2007 ▶); cell refinement: *SAINT* (Bruker, 2007 ▶); data reduction: *SAINT*; program(s) used to solve structure: *SHELXS97* (Sheldrick, 2008 ▶); program(s) used to refine structure: *SHELXL97* (Sheldrick, 2008 ▶); molecular graphics: *ORTEP-3 for Windows* (Farrugia, 1997 ▶); software used to prepare material for publication: *WinGX* (Farrugia, 1999 ▶) and *PLATON* (Spek, 2009 ▶).

## Supplementary Material

Crystal structure: contains datablocks global, I. DOI: 10.1107/S1600536809016900/fj2215sup1.cif

Structure factors: contains datablocks I. DOI: 10.1107/S1600536809016900/fj2215Isup2.hkl

Additional supplementary materials:  crystallographic information; 3D view; checkCIF report

## Figures and Tables

**Table 1:** Hydrogen-bond geometry (Å, °)

*D*—H⋯*A*	*D*—H	H⋯*A*	*D*⋯*A*	*D*—H⋯*A*
N1—H1⋯O3	0.86	1.99	2.644 (2)	132
O4—H4⋯O3^i^	0.82	1.89	2.712 (2)	178
C5—H5⋯O1^ii^	0.93	2.47	3.372 (3)	162
C6—H6⋯O2	0.93	2.50	2.878 (3)	104
C9—H9⋯O4	0.93	2.35	2.700 (3)	102
C11—H11⋯O1^iii^	0.93	2.55	3.429 (3)	159
C12—H12⋯O2	0.93	2.37	3.032 (3)	128
C4—H4*A*⋯*Cg*2^iv^	0.93	2.84	3.765 (3)	174

Symmetry codes: (i) 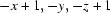; (ii) 
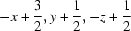
; (iii) 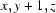; (iv) 
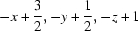
. *Cg*2 is the centroid of the C7–C12 ring.

**Fig. 1.**
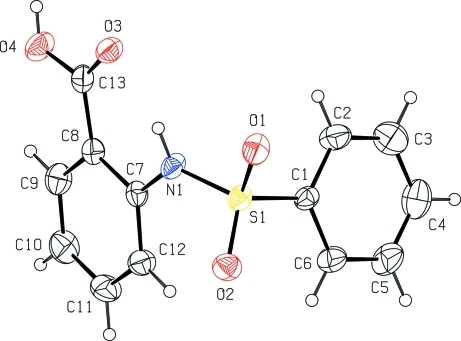
A view of the title compound showing the atom labelling scheme and displacement ellipsoids at the 30% probability level.

**Fig. 2.**
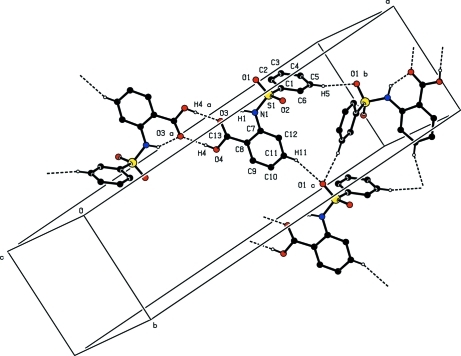
View of the hydrogen bonding interactions of (I) in the unit cell. Hydrogen bonds are indicated by dashed lines and H atoms not involved in the shown interactions have been omitted for clarity.

**Fig. 3.**
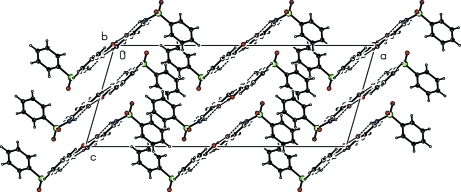
The molecular packing for (I) viewed down the b axis.

## supplementary crystallographic information

### Comment

The title compound is sulfonamide derivative and prepared by the simple condensation of anthranilic acid and benzene sulfonylchloride. The anthranilic sulfonamide derivative has been investigated as an inhibitors of Methionine aminopeptidase-2 (MetAP2), a novel target for cancer therapy (Sheppard *et al.* 2006). The title compound is reported as in countinuation of our studies on the synthesis of sulfonamide derivatives (Arshad *et al.*, 2009).

In the compound, (I), (Fig. 1), the C—O distances are as expected. The S—C_Ph_ distance of 1.763 (2)Å compare well with the literature value of 1.763 (9)Å (Allen *et al.*, 1987). The S1—N1 distance of 1.6213 (18) Å is shorter than the literature value of 1.6458 (11) Å (Wijeyesakere *et al.*, 2008). The mean S=O distance of 1.4248 (16) Å is comparable with the reported value of 1.436 (2)Å (Sethu Sankar *et al.*, 2002). The interplanar angle between the phenyl rings in (I) is 89.01 (12)°. The slight increases in the N1—C7—C8 [119.10 (16)°] and C7—C8—C13 [121.97 (17)°] angles, from the ideal value of 120°, can be attributed to the steric interaction of the N1 and C13 substituents.

The molecular structures are stabilized by intramolecular N—H···O and C—H···O hydrogen bonding interactions. The crystal packing is stabilized by C—H···O and O—H···O hydrogen bonds, and C—H···π interactions (Table 1). The O—H···O hydrogen bond forms a cyclic dimer, with graph-set motif *R*^2^_2_(8), about a centre of symmetry (Fig. 2). Fig. 3 shows the molecular packing for (I) viewed down the *b* axis.

### Experimental

Anthranilic acid (1 g, 7.3 mmol) was dissolved in distilled water (10 ml) in a round bottom flask (25 ml). The pH of the solution was maintained at 8–9 using 1*M*, Na_2_CO_3_. Benzene sulfonylchloride (1.29 g, 7.3 mmol) was then added to the above solution and stirred atroom temperature until all the benzene sulfonyl chloride was consumed. On completion of the reaction the pH was adjusted 1–2, using 1 N HCl. The precipitate obtained was filtered, washed with distilled water, dried and recrystalized in methanol to yield brownish black crystals.

### Refinement

All H atoms are clearly observed using the X-ray difference Fourier maps. H atoms were fixed geometrically and treated as riding, with C—H = 0.93 Å, N—H = 0.86 Å and O—H = 0.82 Å, with *U*_iso_(H) = 1.2*U*_eq_(C,*N*) and *U*_iso_(H) = 1.5*U*_eq_(O).

### Crystal data

**Table:**

C_13_H_11_NO_4_S	*F*(000) = 1152
*M**_r_* = 277.30	*D*_x_ = 1.465 Mg m^−^^3^
Monoclinic, *C*2/*c*	Mo *K*α radiation, λ = 0.71073 Å
Hall symbol: -C 2yc	Cell parameters from 2374 reflections
*a* = 27.271 (3) Å	θ = 2.5–23.4°
*b* = 8.7223 (9) Å	µ = 0.27 mm^−^^1^
*c* = 11.0077 (10) Å	*T* = 296 K
β = 106.149 (3)°	Prism, brownish black
*V* = 2515.0 (4) Å^3^	0.36 × 0.26 × 0.11 mm
*Z* = 8	

### Data collection

**Table:**

Bruker Kappa APEXII CCD area-detector diffractometer	1824 reflections with *I* > 2σ(*I*)
Radiation source: sealed tube	*R*_int_ = 0.047
graphite	θ_max_ = 27.9°, θ_min_ = 2.5°
φ and ω scans	*h* = −35→35
12048 measured reflections	*k* = −11→11
2989 independent reflections	*l* = −14→13

### Refinement

**Table:**

Refinement on *F*^2^	Primary atom site location: structure-invariant direct methods
Least-squares matrix: full	Secondary atom site location: difference Fourier map
*R*[*F*^2^ > 2σ(*F*^2^)] = 0.041	Hydrogen site location: inferred from neighbouring sites
*wR*(*F*^2^) = 0.105	H-atom parameters constrained
*S* = 1.00	*w* = 1/[σ^2^(*F*_o_^2^) + (0.0454*P*)^2^ + 0.631*P*] where *P* = (*F*_o_^2^ + 2*F*_c_^2^)/3
2989 reflections	(Δ/σ)_max_ < 0.001
173 parameters	Δρ_max_ = 0.34 e Å^−^^3^
0 restraints	Δρ_min_ = −0.25 e Å^−^^3^

### Special details

**Table:**

Geometry. Bond distances, angles *etc*. have been calculated using the rounded fractional coordinates. All su's are estimated from the variances of the (full) variance-covariance matrix. The cell e.s.d.'s are taken into account in the estimation of distances, angles and torsion angles
Refinement. Refinement on *F*^2^ for ALL reflections except those flagged by the user for potential systematic errors. Weighted *R*-factors *wR* and all goodnesses of fit *S* are based on *F*^2^, conventional *R*-factors *R* are based on *F*, with *F* set to zero for negative *F*^2^. The observed criterion of *F*^2^ > σ(*F*^2^) is used only for calculating -*R*-factor-obs *etc*. and is not relevant to the choice of reflections for refinement. *R*-factors based on *F*^2^ are statistically about twice as large as those based on *F*, and *R*-factors based on ALL data will be even larger.

### Fractional atomic coordinates and isotropic or equivalent isotropic displacement parameters (Å^2^)

**Table:**

	*x*	*y*	*z*	*U*_iso_*/*U*_eq_	
S1	0.63178 (2)	0.04792 (6)	0.18228 (5)	0.0404 (2)	
O1	0.62113 (5)	−0.11217 (17)	0.16769 (14)	0.0535 (6)	
O2	0.63031 (6)	0.13887 (18)	0.07397 (13)	0.0533 (5)	
O3	0.53363 (5)	0.03052 (18)	0.39985 (13)	0.0483 (5)	
O4	0.50843 (6)	0.2115 (2)	0.51125 (16)	0.0590 (6)	
N1	0.59038 (6)	0.11156 (19)	0.25095 (17)	0.0454 (6)	
C1	0.69269 (7)	0.0708 (2)	0.28988 (18)	0.0392 (7)	
C2	0.70663 (9)	−0.0206 (3)	0.3954 (2)	0.0588 (9)	
C3	0.75548 (12)	−0.0093 (4)	0.4738 (2)	0.0771 (11)	
C4	0.78960 (10)	0.0910 (4)	0.4466 (3)	0.0750 (10)	
C5	0.77546 (9)	0.1822 (3)	0.3430 (3)	0.0721 (11)	
C6	0.72658 (9)	0.1735 (3)	0.2632 (2)	0.0553 (8)	
C7	0.58485 (7)	0.2638 (2)	0.28900 (18)	0.0380 (6)	
C8	0.55742 (7)	0.2911 (2)	0.37784 (18)	0.0399 (7)	
C9	0.55267 (9)	0.4409 (3)	0.4151 (2)	0.0557 (8)	
C10	0.57274 (10)	0.5620 (3)	0.3654 (2)	0.0649 (10)	
C11	0.59832 (10)	0.5339 (3)	0.2762 (2)	0.0581 (9)	
C12	0.60453 (9)	0.3876 (3)	0.2386 (2)	0.0491 (8)	
C13	0.53286 (7)	0.1656 (3)	0.42990 (19)	0.0436 (7)	
H1	0.56950	0.04520	0.26590	0.0540*	
H2	0.68350	−0.08900	0.41350	0.0700*	
H3	0.76540	−0.07010	0.54590	0.0930*	
H4	0.49630	0.13700	0.53800	0.0890*	
H4A	0.82270	0.09660	0.49950	0.0900*	
H5	0.79880	0.25070	0.32560	0.0870*	
H6	0.71670	0.23620	0.19230	0.0660*	
H9	0.53540	0.45970	0.47550	0.0670*	
H10	0.56910	0.66170	0.39160	0.0780*	
H11	0.61160	0.61550	0.24100	0.0700*	
H12	0.62210	0.37080	0.17860	0.0590*	

### Atomic displacement parameters (Å^2^)

**Table:**

	*U*^11^	*U*^22^	*U*^33^	*U*^12^	*U*^13^	*U*^23^
S1	0.0407 (3)	0.0349 (3)	0.0494 (3)	−0.0008 (2)	0.0191 (2)	−0.0065 (2)
O1	0.0497 (9)	0.0363 (9)	0.0768 (11)	−0.0047 (7)	0.0212 (8)	−0.0151 (8)
O2	0.0633 (10)	0.0550 (10)	0.0458 (8)	0.0050 (8)	0.0220 (7)	0.0022 (7)
O3	0.0485 (9)	0.0503 (10)	0.0520 (9)	−0.0019 (7)	0.0236 (7)	0.0001 (7)
O4	0.0627 (11)	0.0616 (11)	0.0651 (10)	0.0020 (9)	0.0383 (8)	−0.0051 (9)
N1	0.0422 (10)	0.0327 (10)	0.0695 (12)	−0.0042 (8)	0.0294 (8)	−0.0062 (9)
C1	0.0426 (11)	0.0361 (12)	0.0444 (11)	−0.0018 (9)	0.0212 (9)	−0.0038 (9)
C2	0.0610 (16)	0.0687 (18)	0.0498 (13)	−0.0095 (13)	0.0208 (12)	0.0077 (12)
C3	0.079 (2)	0.096 (2)	0.0506 (15)	0.0031 (17)	0.0085 (14)	0.0086 (14)
C4	0.0488 (15)	0.091 (2)	0.0768 (19)	−0.0042 (15)	0.0038 (13)	−0.0190 (17)
C5	0.0482 (15)	0.072 (2)	0.096 (2)	−0.0214 (13)	0.0199 (14)	−0.0026 (17)
C6	0.0518 (14)	0.0486 (15)	0.0687 (15)	−0.0099 (11)	0.0221 (12)	0.0039 (12)
C7	0.0347 (10)	0.0343 (11)	0.0439 (11)	0.0032 (8)	0.0091 (8)	−0.0035 (9)
C8	0.0371 (11)	0.0422 (12)	0.0394 (11)	0.0042 (9)	0.0092 (8)	−0.0026 (9)
C9	0.0577 (14)	0.0537 (15)	0.0604 (14)	0.0092 (12)	0.0242 (11)	−0.0096 (12)
C10	0.0754 (18)	0.0400 (14)	0.0830 (18)	0.0097 (13)	0.0280 (14)	−0.0104 (13)
C11	0.0707 (16)	0.0366 (14)	0.0695 (16)	0.0009 (12)	0.0235 (13)	0.0040 (11)
C12	0.0577 (14)	0.0403 (13)	0.0550 (13)	0.0016 (10)	0.0250 (11)	−0.0002 (11)
C13	0.0355 (11)	0.0547 (15)	0.0399 (11)	0.0077 (10)	0.0093 (9)	−0.0007 (11)

### Geometric parameters (Å, °)

**Table:**

S1—O1	1.4262 (16)	C7—C8	1.407 (3)
S1—O2	1.4233 (16)	C8—C13	1.479 (3)
S1—N1	1.6213 (18)	C8—C9	1.386 (3)
S1—C1	1.763 (2)	C9—C10	1.372 (4)
O3—C13	1.226 (3)	C10—C11	1.375 (4)
O4—C13	1.318 (3)	C11—C12	1.367 (4)
O4—H4	0.8200	C2—H2	0.9300
N1—C7	1.413 (2)	C3—H3	0.9300
N1—H1	0.8600	C4—H4A	0.9300
C1—C6	1.376 (3)	C5—H5	0.9300
C1—C2	1.372 (3)	C6—H6	0.9300
C2—C3	1.375 (4)	C9—H9	0.9300
C3—C4	1.370 (5)	C10—H10	0.9300
C4—C5	1.356 (4)	C11—H11	0.9300
C5—C6	1.380 (4)	C12—H12	0.9300
C7—C12	1.388 (3)		
			
S1···H12	2.8300	C13···O3^iv^	3.408 (3)
O1···C13^i^	3.059 (3)	C13···C7^vii^	3.541 (3)
O1···C5^ii^	3.372 (3)	C13···O1^iii^	3.059 (3)
O1···O4^i^	3.197 (2)	C13···N1^vii^	3.429 (3)
O2···C12	3.032 (3)	C6···H3^i^	3.0100
O2···C2^i^	3.396 (3)	C9···H9^viii^	3.1000
O2···O3^i^	3.162 (2)	C11···H4A^x^	3.0100
O3···O2^iii^	3.162 (2)	C12···H4A^x^	3.0100
O3···O4^iv^	2.712 (2)	C13···H1	2.5200
O3···C13^iv^	3.408 (3)	C13···H4^iv^	2.8100
O3···N1	2.644 (2)	H1···O3	1.9900
O4···O3^iv^	2.712 (2)	H1···C13	2.5200
O4···O1^iii^	3.197 (2)	H1···O3^vii^	2.9000
O1···H5^ii^	2.4700	H2···O1	2.7700
O1···H2	2.7700	H2···O2^iii^	2.6200
O1···H11^v^	2.5500	H3···C6^iii^	3.0100
O2···H2^i^	2.6200	H4···O3^iv^	1.8900
O2···H6	2.5000	H4···C13^iv^	2.8100
O2···H10^vi^	2.8200	H4···H4^iv^	2.5600
O2···H12	2.3700	H4A···C11^x^	3.0100
O3···H1^vii^	2.9000	H4A···C12^x^	3.0100
O3···H4^iv^	1.8900	H5···O1^ix^	2.4700
O3···H1	1.9900	H6···O2	2.5000
O4···H10^viii^	2.8500	H9···O4	2.3500
O4···H9	2.3500	H9···C9^viii^	3.1000
N1···O3	2.644 (2)	H9···H9^viii^	2.2600
N1···C13^vii^	3.429 (3)	H10···O4^viii^	2.8500
C2···O2^iii^	3.396 (3)	H10···O2^xi^	2.8200
C5···O1^ix^	3.372 (3)	H11···O1^xii^	2.5500
C7···C13^vii^	3.541 (3)	H12···S1	2.8300
C8···C8^vii^	3.581 (3)	H12···O2	2.3700
C12···O2	3.032 (3)		
			
O1—S1—O2	119.60 (9)	C9—C10—C11	119.0 (2)
O1—S1—N1	103.97 (9)	C10—C11—C12	120.9 (2)
O1—S1—C1	108.11 (9)	C7—C12—C11	120.7 (2)
O2—S1—N1	109.80 (10)	O3—C13—O4	121.7 (2)
O2—S1—C1	107.54 (9)	O3—C13—C8	124.28 (18)
N1—S1—C1	107.23 (9)	O4—C13—C8	114.0 (2)
C13—O4—H4	109.00	C1—C2—H2	121.00
S1—N1—C7	127.27 (14)	C3—C2—H2	121.00
C7—N1—H1	116.00	C2—C3—H3	120.00
S1—N1—H1	116.00	C4—C3—H3	120.00
C2—C1—C6	121.0 (2)	C3—C4—H4A	120.00
S1—C1—C2	119.36 (16)	C5—C4—H4A	120.00
S1—C1—C6	119.58 (15)	C4—C5—H5	120.00
C1—C2—C3	118.8 (2)	C6—C5—H5	120.00
C2—C3—C4	120.5 (3)	C1—C6—H6	120.00
C3—C4—C5	120.5 (3)	C5—C6—H6	120.00
C4—C5—C6	120.1 (3)	C8—C9—H9	119.00
C1—C6—C5	119.2 (2)	C10—C9—H9	119.00
C8—C7—C12	119.04 (18)	C9—C10—H10	120.00
N1—C7—C12	121.84 (19)	C11—C10—H10	120.00
N1—C7—C8	119.10 (16)	C10—C11—H11	120.00
C7—C8—C9	118.47 (18)	C12—C11—H11	120.00
C7—C8—C13	121.97 (17)	C7—C12—H12	120.00
C9—C8—C13	119.55 (19)	C11—C12—H12	120.00
C8—C9—C10	121.8 (2)		
			
O1—S1—N1—C7	178.91 (17)	C3—C4—C5—C6	−0.7 (5)
O2—S1—N1—C7	−52.0 (2)	C4—C5—C6—C1	−0.4 (4)
C1—S1—N1—C7	64.56 (19)	N1—C7—C8—C9	179.46 (19)
O1—S1—C1—C2	−40.9 (2)	N1—C7—C8—C13	−1.8 (3)
O2—S1—C1—C2	−171.34 (17)	C12—C7—C8—C9	−2.3 (3)
N1—S1—C1—C2	70.63 (19)	C12—C7—C8—C13	176.42 (19)
O1—S1—C1—C6	135.31 (17)	N1—C7—C12—C11	179.5 (2)
O2—S1—C1—C6	4.9 (2)	C8—C7—C12—C11	1.3 (3)
N1—S1—C1—C6	−113.14 (18)	C7—C8—C9—C10	1.7 (3)
S1—N1—C7—C8	−161.98 (16)	C13—C8—C9—C10	−177.0 (2)
S1—N1—C7—C12	19.8 (3)	C7—C8—C13—O3	−1.0 (3)
S1—C1—C2—C3	175.4 (2)	C7—C8—C13—O4	−179.52 (18)
C2—C1—C6—C5	1.1 (3)	C9—C8—C13—O3	177.7 (2)
C6—C1—C2—C3	−0.8 (4)	C9—C8—C13—O4	−0.8 (3)
S1—C1—C6—C5	−175.07 (19)	C8—C9—C10—C11	−0.1 (4)
C1—C2—C3—C4	−0.3 (4)	C9—C10—C11—C12	−1.0 (4)
C2—C3—C4—C5	1.0 (5)	C10—C11—C12—C7	0.4 (4)

Symmetry codes: (i) *x*, −*y*, *z*−1/2; (ii) −*x*+3/2, *y*−1/2, −*z*+1/2; (iii) *x*, −*y*, *z*+1/2; (iv) −*x*+1, −*y*, −*z*+1; (v) *x*, *y*−1, *z*; (vi) *x*, −*y*+1, *z*−1/2; (vii) −*x*+1, *y*, −*z*+1/2; (viii) −*x*+1, −*y*+1, −*z*+1; (ix) −*x*+3/2, *y*+1/2, −*z*+1/2; (x) −*x*+3/2, −*y*+1/2, −*z*+1; (xi) *x*, −*y*+1, *z*+1/2; (xii) *x*, *y*+1, *z*.

### Hydrogen-bond geometry (Å, °)

**Table:**

*D*—H···*A*	*D*—H	H···*A*	*D*···*A*	*D*—H···*A*
N1—H1···O3	0.86	1.99	2.644 (2)	132
O4—H4···O3^iv^	0.82	1.89	2.712 (2)	178
C5—H5···O1^ix^	0.93	2.47	3.372 (3)	162
C6—H6···O2	0.93	2.50	2.878 (3)	104
C9—H9···O4	0.93	2.35	2.700 (3)	102
C11—H11···O1^xii^	0.93	2.55	3.429 (3)	159
C12—H12···O2	0.93	2.37	3.032 (3)	128
C4—H4A···Cg2^x^	0.93	2.84	3.765 (3)	174

Symmetry codes: (iv) −*x*+1, −*y*, −*z*+1; (ix) −*x*+3/2, *y*+1/2, −*z*+1/2; (xii) *x*, *y*+1, *z*; (x) −*x*+3/2, −*y*+1/2, −*z*+1.
